# Viability and validity of the bispectral index to measure sleep in patients in the intensive care unit

**DOI:** 10.5935/0103-507X.20200083

**Published:** 2020

**Authors:** Rodolfo Augusto Alves Pedrão, Rodrigo Jardim Riella, Kathy Richards, Silvia Regina Valderramas

**Affiliations:** 1 Intensive Care Unit, Hospital de Clínicas, Universidade Federal do Paraná - Curitiba (PR), Brazil.; 2 Instituto Lactec - Instituto de Tecnologia para o Desenvolvimento - Curitiba (PR), Brazil.; 3 University of Texas - Austin, Texas, United States.; 4 Postgraduate Program in Internal Medicine and Health Sciences and Department of Prevention and Rehabilitation in Physical Therapy, Universidade Federal do Paraná - Curitiba (PR), Brazil.

**Keywords:** Sleep, Sleep initiation and maintenance disorders, Sleep deprivation, Sleep disorders, circadian rhythm, Consciousness monitors, Intensive care units, Sono, Distúrbios do início e da manutenção do sono, Privação do sono, Transtornos do sono do ritmo circadiano, Monitores de consciência, Unidades de terapia intensiva

## Abstract

**Objective:**

To investigate the viability of the bispectral index in the sleep evaluation of critically ill patients and to quantify the associations of sleep parameters measured by this index with the Richards-Campbell Sleep Questionnaire and environmental noise.

**Methods:**

This was a cross-sectional observational study that evaluated critically ill adults with diseases of low or moderate severity. The following were measured: total sleep volume and time, deep sleep volume and time, continuous sleep volume and time, sleep onset latency, and environmental sound pressure level. The subjective perception of sleep was evaluated with the Richards-Campbell Sleep Questionnaire the morning after each night of observation.

**Results:**

Patients had a low total sleep time (234 minutes), a predominance of superficial sleep stages, and little deep sleep (1.7 minutes). The total, deep, and continuous sleep volumes were 3,679, 9.4, and 3,143 (bispectral index units × minutes), respectively. The sleep latency was 94 minutes. The mean score of the Richards-Campbell Sleep Questionnaire was 57.9. Total sleep volume, total sleep time, and continuous sleep volume were weakly correlated with the Richards-Campbell Sleep Questionnaire depth of sleep domain score, overall sleep quality domain score, and total score. Total volume, total time, and continuous volume were moderately correlated with the occurrence of awakenings domain score.

**Conclusion:**

The bispectral index is an instrument with limited viability to monitor the sleep of lucid patients and patients with low to moderate disease severity in the intensive care unit. Patients with higher total sleep volume, total sleep time, and continuous sleep volume had better overall sleep perception.

## Abstract (pt)

**Objetivo:**

Investigar a viabilidade do índice bispectral na avaliação do sono de pacientes criticamente enfermos lúcidos e a associação entre os parâmetros do sono aferidos por esse índice, pelo Questionário de Sono de Richards-Campbell e pelo ruído ambiental.

**Métodos:**

Estudo observacional transversal que avaliou indivíduos adultos criticamente enfermos com doenças de gravidades baixa ou moderada. Foram aferidos: volume e tempo total de sono, volume e tempo de sono profundo, volume e tempo de sono contínuo, latência para o início do sono e pressão sonora ambiental. A percepção subjetiva do sono foi registrada com o Questionário de Sono de Richards-Campbell nas manhãs subsequentes às noites de observação.

**Resultados:**

Os pacientes apresentaram tempo total de sono reduzido (234 minutos), predominância dos estágios superficiais e pouco sono profundo (1,7 minutos). Os volumes de sono total, profundo e contínuo foram 3.679, 9,4 e 3.143 índice bispectral versus minutos, respectivamente. A latência para o sono foi de 94 minutos. O escore médio do Questionário de Sono de Richards-Campbell foi 57,9. Foram observadas correlações de magnitudes fracas entre volume total, tempo total, volume contínuo e os domínios do Questionário de Sono de Richards-Campbell profundidade do sono, qualidade geral de sono e escore total; e correlações de magnitudes moderadas entre volume total, tempo total, volume contínuo e o domínio ocorrência de despertares.

**Conclusão:**

O índice bispectral é um instrumento com viabilidade limitada para monitorar o sono de pacientes lúcidos e com enfermidades de gravidades baixa e moderada na unidade de terapia intensiva, e pacientes que apresentaram maior volume, tempo total e volume de sono contínuo tiveram melhor percepção global do sono.

## INTRODUCTION

The sleep of critically ill patients is usually poorly restorative, superficial,^(1)^ and fragmented.^(2-4)^ Sleep deprivation is a major cause of stress and anxiety in patients discharged from the intensive care unit (ICU)^(5,6)^ and is associated with deleterious outcomes, both during hospitalization and after hospital discharge.^(7,8)^

Although necessary, the study of sleep in the ICU is challenging. Polysomnography, the gold-standard method of evaluation, is expensive and difficult to perform in the ICU and should be interpreted with caution due to the effects of disease and of treatment, especially in patients with more severe conditions.^(9)^ Accessible and easier-to-use alternatives have been sought in the form of objective (bispectral index - BIS and actigraphy) and subjective (questionnaires) sleep assessment instruments,^(10)^ but the practical applicability of these strategies in the ICU still need to be corroborated.

The BIS is calculated by the automatic processing of electroencephalographic data and has great potential for the investigation of sleep in the ICU because it is simpler and more accessible than polysomnography. Although the correlation between BIS and sleep is well defined in the experimental setting,^(11)^ only two studies have used BIS monitoring systems to assess sleep in the ICU.^(12,13)^

The Richards-Campbell Sleep Questionnaire® (RCSQ®) is a widely available, easily applicable instrument with excellent internal consistency and moderate correlation with polysomnography,^(14,15)^ and it has been cross-culturally translated into Brazilian Portuguese.^(16)^

Among the various factors that affect sleep in the hospital environment, environmental noise is considered one of the most influential,^(4,17,18)^ and it is also associated with the occurrence of delirium.^(19,20)^

In this study, we investigated the viability of BIS in the evaluation of sleep in critically ill patients and the associations between sleep parameters measured by this index, the parameters measured by the RCSQ, and environmental noise.

## METHODS

This cross-sectional observational study was conducted in an eight-bed surgical ICU of a tertiary public hospital. It was conducted in accordance with the 1975 Declaration of Helsinki (revised in 2000), approved by the institutional Research Ethics Committee (registration number 83082118.7.0000.0096), and registered in the Brazilian Registry of Clinical Trials (*Registro Brasileiro de Ensaios Clínicos* - REBEC; record number RBR-2ss28r). All participants gave written consent to participate before the start of monitoring.

There was no active sleep promotion protocol at the time of data collection, and no participant used earplugs or eye masks. The study included adult patients of both sexes who were treated for diseases of low or moderate severity, who had the prospect of staying the following night in the ICU, and who were sufficiently lucid to understand and accept the terms of the free and informed consent form. Patients undergoing surgical procedures were included on the day following the intervention to minimize the effect of anesthetics on sleep. Patients under residual effects of sedatives (with score <0 on the Richmond Agitation and Sedation Scale), who reported moderate or high pain intensity (according to the visual analog scale),^(21)^ who showed discomfort with the BIS sensors, who had clinical deterioration that compromised their ability to understand and accept the terms of the free and informed consent form, whose data could not be recorded throughout the night, or who experienced delirium as identified by the Confusion Assessment Method for Intensive Care Unit (CAM-ICU) instrument^(22)^ were excluded from the study.

Data were collected from August 2018 to June 2019. Patients in our ICU were screened for study eligibility every day of the week. Only one patient was monitored each night because there was only one BIS monitoring system available for the study. All data were collected by the same researcher.

After recruitment, the demographic, anthropometric, and clinical data of the participants were recorded. The severity of the disease presented by the individual was estimated on the day of monitoring with the Acute Physiology and Chronic Health Evaluation II (APACHE II) index.^(23)^

A BIS Quatro sensor (Covidien LLC, Mansfield, United States) was placed on the left frontal region of the participant’s body and was connected to the BIS™ Vista Monitoring System (Covidien LLC, Mansfield, United States) from 7:00 pm to 7:00 am the following morning. The BIS values and their respective signal quality were recorded every minute during the observation period. Immediately after placement of the head sensor, the perception of pain and discomfort due to the presence of the BIS sensor was again evaluated.

The parameters recorded by the BIS were total sleep volume, total sleep time, deep sleep volume, deep sleep time, continuous sleep volume, continuous sleep time, and sleep onset latency. Sleep duration was expressed in minutes and sleep volume as a function of the area under the BIS curve *versus* time (in minutes).^(13)^ We adopted a BIS value < 80 as the onset of superficial sleep and < 40 as the onset of deep sleep.^(24-26)^ Sleep continuity was recorded considering periods of uninterrupted sleep ≥ 10 minutes as significant.^(27,28)^ The sleep onset latency, i.e., the time from the start of monitoring to sleep stage 1,^(29)^ was expressed in minutes.

The environmental sound pressure level was recorded from 7:00 pm to 7:00 am the following morning with a professional class 1 sound level meter^(30)^ with a datalogger function (model DT-8852, CEM Instruments, India). This instrument was subjected to certified calibration and placed 1 m from the headboard of the patient’s bed. The sound capture frequency was 2 seconds. The capture unit used was decibels (dB). The compensation circuit was set to slow *A* weighting. The *A*-weighted equivalent continuous noise levels (LAEq) of the observation periods^(10)^ were calculated.

In the morning after each monitoring night, the Brazilian Portuguese version of the RCSQ was applied to the patient. The RCSQ has domains that assess perceptions of depth of sleep, sleep onset latency, occurrence of awakenings, ability to return to sleep after awakening, and overall sleep quality. Each domain was scored by the evaluated individual on a visual analog scale ranging from zero to 100mm, with higher scores indicating greater satisfaction. The total score, i.e., the mean of the five domains, represented the overall perception of sleep.^(15)^

### Statistical analysis

The assumption of normality of the variables was tested with the Shapiro-Wilk test, and the results are presented as median and 25^th^ - 75th percentile, mean and standard deviation, or frequency, depending on the type of variable and the data distribution. The Spearman correlation coefficient was used to examine the degrees of association between the BIS parameters, the RCSQ total score, and domain scores and the environmental sound pressure level. The magnitude scale proposed by Batterham and Hopkins^(31)^ was used to interpret the correlations, with < 0.1 considered trivial; 0.10 - 0.29, small; 0.30 - 0.49, moderate; 0.50 - 0.69, large; 0.70 - 0.90, very large and > 0.90, almost perfect.

Data were analyzed in the Statistical Package for the Social Sciences (IBM), version 22. The significance level was set at 5%.

## RESULTS

After screening, 49 individuals met the inclusion criteria. However, seven individuals refused to participate; one showed rapid clinical improvement and was discharged from the ICU; two had delirium and could not be monitored; five dropped out of the study after technical failure of sleep monitoring or because of discomfort with the BIS sensor; and five others underwent procedures that made monitoring impossible. Thus, 29 participants completed the monitoring. Table 1 summarizes the characteristics of the study patients. All were right-handed.

**Table 1:** Demographic, anthropometric, and clinical characteristics of patients who completed the night of monitoring

Variable	
Age (years)	62 (51 - 71.5)
White	26 (89)
Mixed race	3 (11)
Female	14 (48)
APACHE II	10 (7 -13)
Current smoking	1 (3.4)
Current drinking	2 (6.8)
Use of invasive mechanical ventilation	3 (10.3)
Use of noninvasive mechanical ventilation	2 (6.8)
Use of tracheostomy	1 (3.4)
Use of opioids	9 (31)
Use of vasoactive drugs	15 (51.7)
Reason for hospitalization	
Gastrointestinal surgery	16 (55)
Thoracic surgery	3 (10.3)
Urological surgery	2 (6.8)
Pulmonary thromboembolism	2 (6.8)
Liver transplantation	1 (3.4)
Oral and maxillofacial surgery	1 (3.4)
Head and neck surgery	1 (3.4)
Orthopedic surgery	1 (3.4)
Leptospirosis	1 (3.4)
Myasthenia gravis	1 (3.4)

APACHE II - Acute Physiology and Chronic Health Evaluation II. The results are expressed as median (25th-75th percentile), n (%), or mean (standard deviation).

The measurements using the BIS (Table 2) showed that this group of individuals had low total sleep time (mean of 234 minutes per night of observation) and practically did not reach deep sleep (mean of 1.7 minutes per night of observation), with a mean sleep onset latency of 94 minutes.

**Table 2:** Sleep parameters evaluated by the bispectral index, environmental sound pressure level, pain perception (visual analog scale), and Richards-Campbell Sleep Questionnaire domain scores and total score

Variable	
Sleep parameters (BIS)	
Total volume	3.679 (2.735.28)
Total time	234 (114.74)
Deep volume	9.4 (22.07)
Deep time	1.7 (3.7)
Continuous volume	3.143 (2.571.5)
Continuous time	174 (117.2)
Latency	94 (103.75)
Environmental sound pressure level	
LAEq	57.6 (2.9)
Pain (VAS)	
Intensity	1.73 (2.22)
RCSQ domains	
Depth	48.68 (32.8)
Latency	46.63 (31.83)
Occurrence of awakenings	63.49 (30.67)
Ability to return to sleep	65.11 (30.38)
Overall quality	65.2 (27.34)
Total RCSQ	57.9 (21.75)

BIS - bispectral index; LAEq - A-weighted equivalent continuous noise level (in decibels); VAS - visual analog scale; RCSQ - Richards-Campbell Sleep Questionnaire. Values are expressed as mean (standard deviation). Sleep time and latency expressed in minutes; sleep volume expressed in (bispectral index units <80) × time.

The application of the RCSQ (Table 2) showed that the monitored individuals considered their sleep satisfactory, especially on the domains occurrence of awakenings, ability to return to sleep, and overall sleep quality.

We recorded high levels of noise on the nights of observation (57.6 dB mean LAEq) (Table 2).

**Figure 1:**
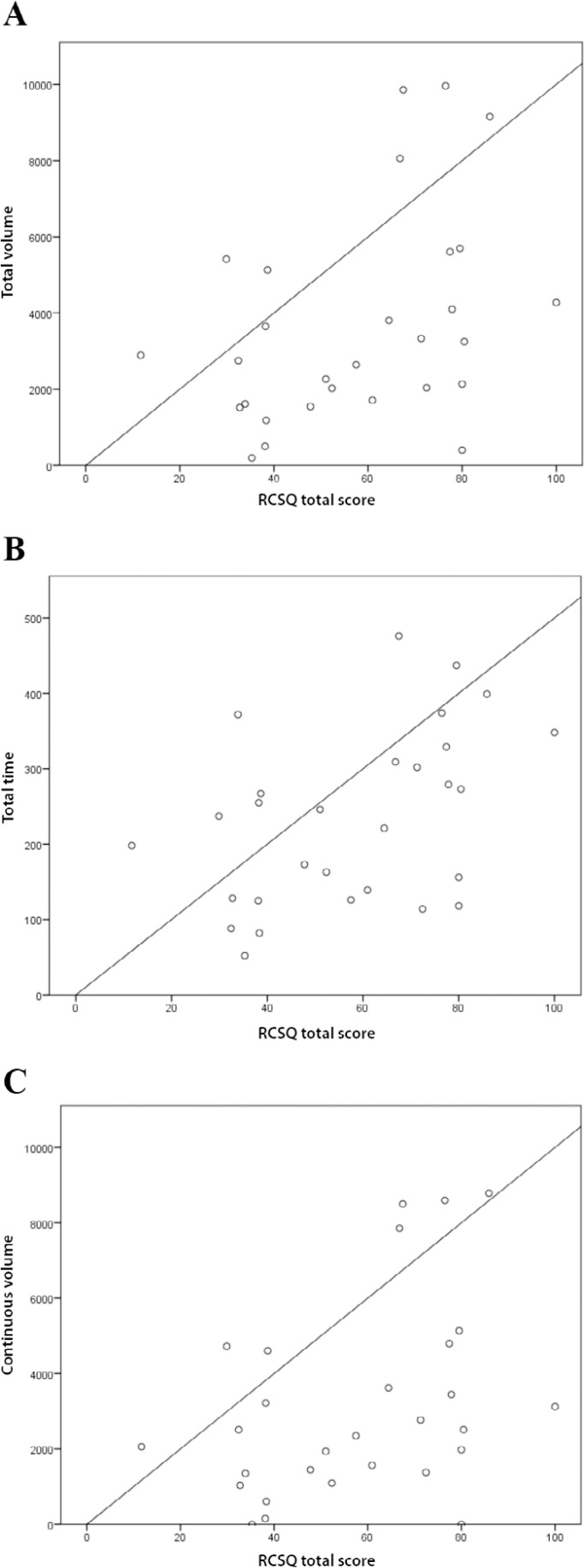
Correlations between three parameters of the bispectral index and the Richards-Campbell Sleep Questionnaire total score. (A) Total volume *versus* total score, (B) total time *versus* total score, and (C) continuous volume *versus* total score. RCSQ - Richards-Campbell Sleep Questionnaire. Total volume

The BIS parameters total volume, total time, and continuous volume were weakly correlated with the RCSQ domains depth of sleep and overall sleep quality and the RCSQ total score. Total volume, total time, and continuous volume were moderately correlated with the occurrence of awakenings domain (Table 3). Figure 1 illustrates the correlations between the parameters total volume, total time, and continuous volume and the RCSQ total score.

**Table 3:** Correlation coefficients (Spearman's rho) between measured sleep parameters (bispectral index), Richards-Campbell Sleep Questionnaire domain

	Total volume	Total time	Deep volume	Deep time	Continuous volume	Continuous time	Latency
Sleep depth	0.47*	0.37*	0.18	0.18	0.43*	0.36	0.33
Sleep onset latency	-0.96	0.03	0.04	-0.27	-0.09	-0.06	0.09
Occurrence of awakenings	0.53†	0.51†	0.32	0.31	0.51†	0.48*	0.34
Ability to return to sleep	0.26	0.35	0.17	0.18	0.28	0.30	0.21
Overall quality	0.46*	0.43*	0.06	0.07	0.43*	0.40*	0.30
Total RCSQ	0.39*	0.44*	0.25	0.22	0.38*	0.35	0.35
LAEq	0.12	-0.20	-0.18	-0.21	0.16	-0.10	0.10

RCSQ - Richards-Campbell Sleep Questionnaire; LAEq - A-weighted equivalent continuous noise level.

* p < 0.05;

† p < 0.01.

## DISCUSSION

The results of this study demonstrate that sleep monitoring in lucid ICU patients through BIS revealed reduced sleep time, with a predominance of superficial stages and virtually no deep sleep. In addition, weak correlations were observed between total volume, total time, and continuous volume and some RCSQ scores (depth of sleep, overall quality of sleep, and total score). Correlations of moderate magnitude were found between total volume, total time, and continuous volume and the occurrence of awakenings domain.

The low total sleep time and the superficiality of the sleep stages corroborate the findings of other studies^(10,32)^ and the concept that ICU stay is associated with sleep deprivation and its deleterious consequences.^(19,33,34)^ Regarding the reliability of the capture quality and the signal recording by the BIS in the ICU, it should be noted that previous studies used the A-1000 monitoring systems^(12)^ and BIS-XP,^(13)^ and this study used the BIS™ Vista Monitoring System. This system has a more state-of-the-art algorithm, lower potential for interference by electromyographic activity, and better signal capture quality.^(35)^ The analysis of the BIS values and the quality of the signal of this index over time showed a negative correlation between the two, with a gradual improvement of this index as the sleep deepened. Therefore, the signal quality was invariably high during sleep, which reinforces the adequacy of the BIS for such records. In addition, the use of the area under the curve to calculate the total and continuous sleep volumes allowed us to estimate the amount and quality of sleep,^(13)^ expanding the interpretation of BIS information beyond the mere analysis of sleep time.

We believe that the weak associations found between the parameters evaluated by the BIS and some of the RCSQ domains may be at least partially explained by the overestimation of sleep quality usually observed with subjective instruments, such as the RCSQ.^(1,36)^

We found no correlation between sleep onset latency and BIS measurements. The ritual of preparing for sleep (turning off the lights, changing from standing to supine, and relaxing) induces physiological changes that trigger sleep.^(37)^ In the ICU, this ritual does not occur, limiting the importance of the analysis of sleep onset latency in the evaluation of subjective sleep quality.

Although the study included individuals who underwent major surgery, their reported pain intensity was low. We believe that this was true because the patients undergoing surgical procedures were included into the study only on the day after the surgical intervention, which enabled the use of adequate analgesia beforehand.

The environmental sound pressure exceeded the 35dB recommended by the World Health Organization (WHO),^(38)^ but the sound level had no correlation with sleep parameters. Other studies have also concluded that, although important, environmental noise is only one of the myriad of factors involved in sleep,^(39,40)^ which may also have been true in this study.

Although this is the largest known study using BIS to investigate sleep in the ICU, some limitations should be considered. Part of the sleep of critically ill patients occurs during the day,^(1,10,32)^ but we monitored only the nocturnal period. There were losses to follow-up due to displacement of or discomfort with the sensor (n = 5); however, all patients were lucid and had no residual effect of sedatives, unlike patients in whom BIS is usually used for anesthesia monitoring. Even though there is intra- and interindividual variability in the sleep of critically ill patients^(1)^ and we do not know if the first-night effect^(41)^ occurs with BIS, each participant was monitored only for one night, and these results are only valid for lucid patients with diseases of low or moderate severity. Although this selection criterion limited the generalization of the results, it ensured the reliability of the measurements because the evaluation of sleep in agitated, clinically unstable, or sedated patients is not feasible or is at best biased.^(9,42,43)^

## CONCLUSION

The bispectral index is an instrument with limited feasibility to monitor the sleep of lucid patients and patients with low to moderate disease severity in the intensive care unit. Patients with higher total sleep volume, higher total sleep time, and greater continuous sleep volume had better overall sleep perception. More studies using bispectral index are needed to explore the full potential of this accessible technology in sleep monitoring in the intensive care unit.
